# Consideration in Randomized Placebo-Controlled Trial on Neck Pain to Avoid the Placebo Effect in Analgesic Action

**DOI:** 10.3389/fphar.2022.836008

**Published:** 2022-05-19

**Authors:** Yue-Li Sun, Min Yao, Yue-Feng Zhu, Meng-Chen Yin, Jin-Tao Liu, Xin Chen, Jin Huang, Yu-Xiang Dai, Wen-Hao Wang, Zeng-Bin Ma, Yong-Jun Wang, Xue-Jun Cui

**Affiliations:** ^1^ Longhua Hospital, Shanghai University of Traditional Chinese Medicine, Shanghai, China; ^2^ Spine Institute, Shanghai University of Traditional Chinese Medicine, Shanghai, China; ^3^ Key Laboratory of the Ministry of Education of Chronic Musculoskeletal Disease, Shanghai, China; ^4^ Huadong Hospital, Fudan University, Shanghai, China; ^5^ Institutes of Integrative Medicine, Fudan University Institute of Geriatrics and Gerontology, Shanghai, China; ^6^ Suzhou TCM Hospital, Nanjing University of Chinese Medicine, Suzhou, China; ^7^ Gansu University of Traditional Chinese Medicine, Lanzhou, China; ^8^ Affiliated Hospital of Gansu University of Traditional Chinese Medicine, Lanzhou, China; ^9^ Gansu Provincial Hospital of TCM, Lanzhou, China; ^10^ Beijing Hospital, Lanzhou, China

**Keywords:** placebo and nocebo effects, clinical trial, logistic regression, cervical radiculopathy, neck pain

## Abstract

**Background:** In neck pain treatment, many therapies are focused on etiology, while it is well-known that placebo analgesia is also present in these therapies. The specific efficacy for etiology may be underestimated by ignoring their actual placebo effect. In this study, a logistic regression analysis is used to explore the risk factors causing different placebo responses in patients with neck pain among two RCTs. The probability of the placebo effect is predicted based on these risk factors.

**Methods:** Trial A and Trial B were similarly designed, randomized, double-/single-blind, placebo-controlled trials in patients treating neck pain with Qishe pill or Shi-style manipulation. Both studies set a placebo pill twice a day or traction for every other day as control. For further analyses on the placebo effect in neck pain management, logistic regression was used to assess subgroup-placebo interactions. The odds ratio assessed a significant influence on the placebo effect.

**Results:** In this pooled analysis, the total number of patients recruited for these two studies was 284, of which 162 patients received placebo treatment (placebo drug or traction for every other day). No statistically significant differences are found at baseline between the participants with placebo effect and non-placebo effect in the gender, age, and disease duration except in VAS and NDI at the initial time. There are numerically more patients with placebo effect in the shorter disease duration subgroup (< 4 months [76%]), higher initial VAS subgroup (>60 mm [90%]), and worse initial NDI subgroup (>24 [72%]) compared with the gender and age subgroup. An ROC curve is established to assess the model-data fit, which shows an area under the curve of 0.755 and a 95% confidence interval of 0.677–0.830. Participants who show placebo effect after 2 weeks have significantly lower VAS scores after 4 weeks, while there is no significant difference in NDI improvement between the two groups after 4 weeks.

**Conclusion:** Neck pain patients with shorter disease duration are more likely to overscore their pain severity, because of their less experience in pain perception, tolerance, and analgesia expectation.

## Introduction

Neck pain is known as pain in the neck with or without upper limb disorders, which may last for several days (Hoy et al., 2014; Safiri et al., 2020). Neck pain has been considered the fourth most common cause of disability in the United States, according to the Global Burden of Disease 2010 study (Safiri et al., 2020). Notably, women are more likely to experience neck pain, with peak prevalence occurring in middle age (Fejer et al., 2006; Hogg-Johnson et al., 2008). Neck pain can be classified by etiology as mechanical, neuropathic, or secondary (such as pain from cardiac or vascular disease). Most acute neck pain episodes resolve with or without treatment, but nearly 50 percent continue to experience some degree of pain or occur frequently (Cohen, 2015; Fehlings et al., 2021).

In neck pain treatment, many therapies are more focused on etiology, such as nerve root compression, muscle spasm, inflammation, and so on, while it is well-known that placebo analgesia also induces discrete physiological changes mediated by the endogenous opioids system, which is called as placebo effect (Benedetti et al., 2005; Sauro and Greenberg. 2005; Tracey. 2010; Carlino et al., 2011; Benedetti. 2013), but its specific efficacy on etiology of neck pain is limited. Therefore, most of the RCTs for pain treatment were designed to evaluate the specific etiological efficacy by comparing with a placebo.

Regarding therapies such as placebo, there are various forms such as sugar pills, saliva injection, or sham surgeries (Harrington, 1997; Finniss, 2013; Benedetti. 2014), which are typically conceptualized as inactive treatments that are used as controls for the active treatment. The placebo effect is about how patients perceive and experience the treatment through their senses, and actively incorporate their previous experiences and current expectations with it (Vase et al., 2014; Shaibani et al., 2017). However, there are currently several challenges and questions on some of the main concerns of the placebo group in RCTs (Rief et al., 2011; Enck et al., 2013; Vase et al., 2015a; Benedetti et al., 2016; Blease et al., 2017). Many meta-analyses have been done over the years to study the extent of placebo responses in RCTs, whose results have been contradictory while some have positive results (Walsh et al., 2002; Silberman, 2009; Hauser et al., 2011; Tuttle et al., 2015) and some have negative results (Vase et al., 2015b).

RCTs for pain treatment are commonly used placebo to identify the specific efficacy of the placebo effect, in which all the settings, providers, and subjects’ baseline are designed with the same and homogenous conditions to ensure comparable as possible (Trochim and Donnelly. 2001). However, the specific efficacy for etiology may be underestimated by ignoring their actual placebo effect. To detect placebo response, some approaches were used in the previous studies (Vase et al., 2002; Vase et al., 2009; Simpson et al., 2014; Gerdesmeyer et al., 2017), including identifying patients’ disease histories, increasing sample size, or setting a non-treatment group. However, these methods were still hard to achieve the purpose of reducing the placebo response in comparison (Simpson et al., 2014). It is evident that many variables could affect the extent of the placebo effect (Vase et al., 2002; Vase et al., 2009) and it is related to how patients perceive the therapeutic intervention (Vase et al., 2014; Shaibani et al., 2017).

Our research team has conducted two randomized placebo-controlled trials on neck pain. To further investigate the analgesic effect of the placebo treatment itself, we combined all the participants’ data in the respective placebo treatment group of these two RCTs for a pooled analysis. Patients receiving placebo treatment were divided into two groups according to self-reported pain relief after 2 weeks. We hypothesized that considerable pain relief in the placebo treatment group may exist placebo effect. Therefore, in this study, a logistic regression analysis was used to explore the risk factors causing different placebo responses in patients with neck pain among two retrospective, blind, placebo-control RCTs. The probability of the placebo effect was predicted based on the risk factors.

## Methods

### Patients and Study Design

Detailed design and clinical results of Trial A and Trial B have been reported (Cui et al., 2013; Yao et al., 2020).

Trial A and Trial B shared many study design elements, allowing for integrated analyses. Briefly, both trials were randomized, blind, placebo-controlled, Phase 2 studies. These studies were conducted in accordance with the principles of the Declaration of Helsinki. The institutional review board or independent ethics committee at each site approved the protocols, and all patients provided written informed consent. All authors had access to the study data and have reviewed and approved the final manuscript.

Trial A and Trial B were conducted in patients diagnosed with cervical spondylotic radiculopathy (CSR), with the primary objective of evaluating the efficacy of Qishe pill (3.75 g, twice a day, bid) or Shi-style manipulation (60 min, every other day, qod) versus placebo (3.75 g, twice a day, bid) or traction (60 min, every other day, qod) as measured by the proportion of patients who became neck pain-free and most neck-related hypofunction at 4 weeks. The placebo and active treatment arms received either the same strength of Qishe pill/shi-style manipulation or placebo/traction (1:1 ratio).

### Inclusion/Exclusion Criteria

All participants were selected from the general outpatient clinic in the five hospitals across China (Longhua Hospital, Shanghai University of TCM; Huadong Hospital, Fudan University; Affiliated Hospital of the Changchun University of TCM; Gansu Provincial Hospital of Traditional Chinese Medicine; and Suzhou TCM Hospital Affiliated to the Nanjing University of Chinese Medicine), where the patients will be diagnosed with cervical radiculopathy confirmed by a neurologist, and then diagnosed based upon clinical symptoms, physical examination on nerve signs, and imaging (Carette and Fehlings. 2005).

Inclusion criteria are age between 18 and 65 years, pain or stiffness in the neck for at least 2 weeks, neck symptoms reproducible during physical examination, and neck pain on neck disability index (NDI) of 18 or more. Further inclusion prerequisites are willingness for treatment and to adhere to measurement regimens, no involvement in litigation, and written informed consent. Excluded criteria are patients whose history, signs, and symptoms suggested a potential non-benign cause (including previous neck surgery) or evidence of a specific pathologic condition.

### Details of Placebo Intervention

At randomization, patients will be assigned to receive Qishe pills (or matching placebo) at 3.75 g twice per day. In this study, the same outer packing will be used for both the Qishe pill and the matching placebo. Therefore, the treating physicians, participants, and investigators will be blinded to treatment assignment.

In Trial A, the placebo is composed of β-cyclodextrin, colorants, and food flavoring, to look and taste similar to the Qishe pill, which is a thin 0.15 g film-coated pill with a slight odor and bitter taste. The Qishe pill and matching placebo used in the trial are both manufactured and provided by a pharmaceutical company that meets the requirements of Good Manufacturing Practice. All significant medicine information, including ingredient composition, heavy metals, etc., are provided by the same company.

In Trial B, patients in the traction treatment group received intermittent cervical traction (ICT) for 20 min, every other day for 4 weeks. A previous Cochrane systematic review reported that mechanical traction presents little effect on neck pain, while it is still believed to be clinically effective (Graham et al., 2008). As a result, we put this control group as a placebo control. Traction was given in supine lying, as this is one of the most comfortable positions during cervical traction. Parameters of intermittent cervical traction: traction poundage ranging from 10 to 20% of the patient’s body weight, holding time: 10–25 s; resting time: 20–50% of holding; resting poundage: 20–40% of traction poundage. The exact parameters (within the above range) were determined by the treating physiotherapist, who had at least 5 years of clinical experience in treating patients with neck pain.

### Study Evaluations and Analyses

The primary study outcome is pain severity (measured with a visual analog scale, VAS). Secondary outcomes are a composite of functional status (measured by NDI), patient satisfaction, and adverse events as reported in the trial. The visual analog scale measures the amount of pain, which is a pain score ranging from 0 (no pain) to 100 mm (very severe pain) (Gould et al., 2001).

Operationally, the VAS score is displayed as a horizontal line, 100 mm in length, with word descriptors anchored at each end. The patient marks on the line the point that they feel represents their perception of their current pain. The VAS score is then determined by measuring in millimeters from the left end of the line to the point that the patient marks. The VAS score and NDI will be measured at all the measurement points (baseline, 2 and 4 weeks of treatment duration). Patients were not shown their previous scores when they went for the current assessment.

### Statistical Analysis

Data handling rules and full analysis methods were previously described in Cui et al. (2013) and Yao et al. (2020). Standard statistical techniques will be used to describe the characteristics of patients in both groups. We will compare baseline characteristics in both groups and if incomparability appears, we will perform the secondary analysis, adjusting for differences. The primary outcome, the amount of pain, will be compared between both groups using analysis of variance for repeated measures in each visit (week2 and week4). Efficacy analyses were conducted in the modified Intent-to-Treat population consisting of patients who took placebo treatment for 4 weeks and had at least 2 post-dose efficacy assessments after 2 and 4 weeks. For the analyses of active placebo effect freedom in subgroups by age, disease duration, and severity, the *p*-value was calculated for placebo-by-subgroup interaction, based on logistic regression with terms for study, subgroup, and placebo-by-subgroup in the model. The odds ratio assessed a significant influence on the placebo effect. The odds ratio (OR) and the flag of *p*-value > 0.05 Hosmer and Lemeshow test for homogeneity of OR are displayed. ORs were created with the probability of the active placebo effect as the numerator and the actual incidence of the active placebo effect as the denominator.

To detect patients who are easy to respond to placebo therapies, a predictive model was constructed based on the multivariable logistic regression coefficients. The predictive performance of the model was quantified with the area under the receiver operating characteristic curve (AUC), which ranges from 0.5 to 1.0 for sensible models. The internal validity of the model was assessed by bootstrapping techniques (Steyerberg, 2008). Model performance in the validation set was quantified with respect to discrimination (AUC) and calibration. Calibration was assessed graphically by plotting observed frequencies against predicted probabilities.

Tests with 2-sided *p*-values less than 0.05 are referred to as having statistical significance for a factor difference unless otherwise noted. However, *p*-values should not be overinterpreted for covariate analyses.

## Results

### Baseline Patient Characteristics

**FIGURE 1:**
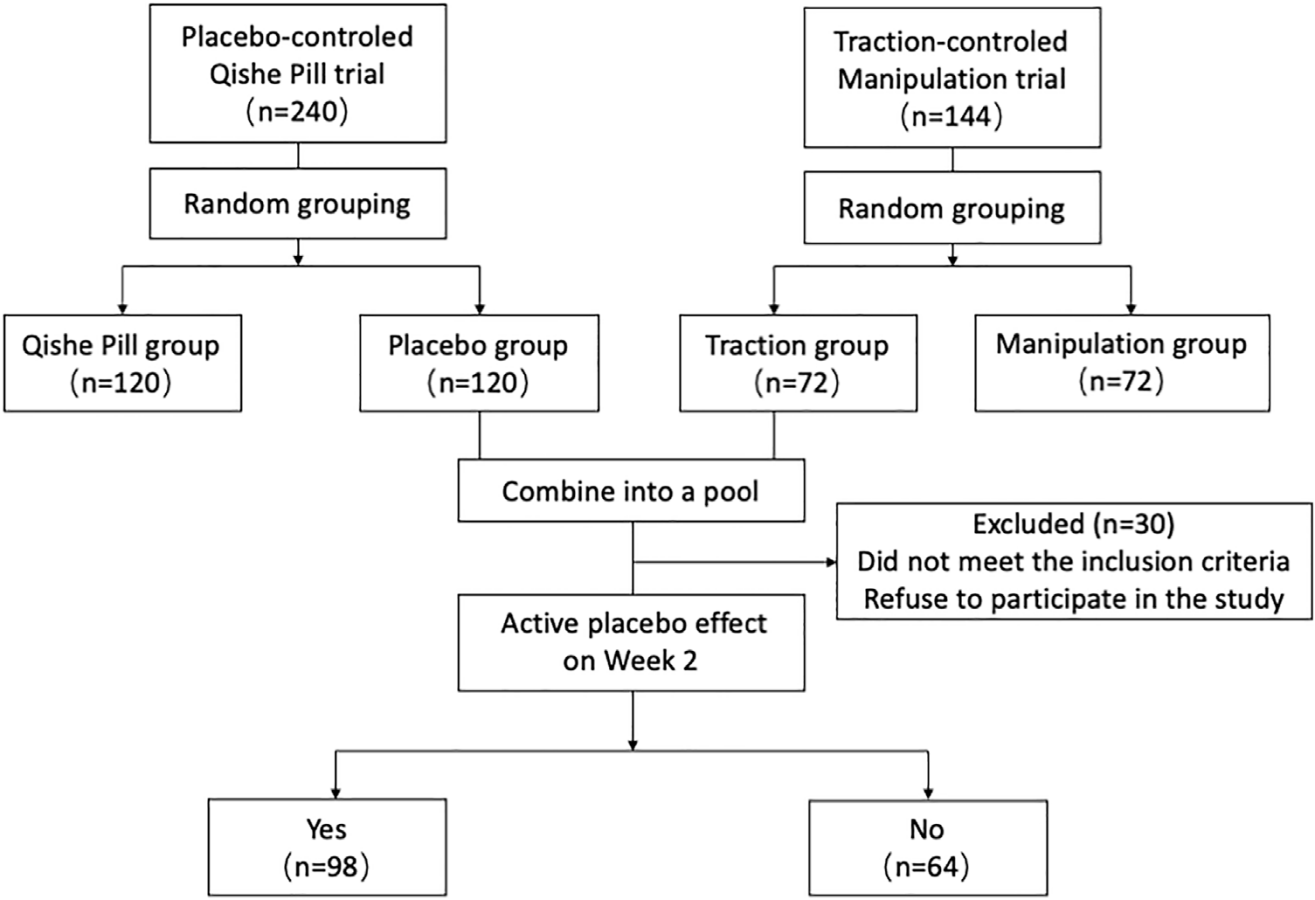
Participant flow and pooled analysis procedure.

The total number of patients recruited for these two studies was 284. The details on patient recruitment, follow-up, and selection during the posthoc analysis are summarized in Figure 1. Thirty participates’ withdrawal (17 in Qishe pill placebo; 13 in traction placebo) included insufficient time, concurrent treatment, and non-conformance to the pooled analysis criteria. Finally, there are 102 participates in the Qishe pill placebo group and 60 participants in the traction placebo group.

Demographics of the patients at baseline are described in Table 1, which shows median values and quartile (25%, 75%) of the age, disease duration, VAS, and NDI.

**TABLE 1:** Summary of demographic information of the patients in different placebo effects.

		Total	Active Placebo Effect	Non-Placebo Effect	*p-*Value
Patient		162	98	64	-
Sex (n[%])	Male	41 [25.31%]	26 [26.53%]	15 [23.43%]	0.714
	Female	121 [74.69%]	72 [73.47%]	49 [76.56%]	
Age (years)		52 (41–60)	52 (39–59)	54 (44.25–60)	0.190
Duration (months)		4 (2–8)	4 (2–8)	5 (2–10)	0.106
VAS (mm)		60 (43–70)	61 (52–72.5)	49.5 (40–60)	0.000^*^
NDI		24 (18–30)	22 (20–32)	22 (17–28)	0.046^*^

^*^, at the p<0.01 level (two-tailed), the difference between groups is significant.

No statistically significant differences were found at baseline between the participates with active placebo effect and non-placebo effect in the gender (*p* > 0.05), age (*p* > 0.05) and disease duration (*p* > 0.05) except in VAS and NDI at the initial time (*p* < 0.05).

### Likely Active Placebo Effect Incidence by Patient Demographic Categories

#### Assessment of Model-Data Fit

Table 2 shows the active placebo effect probability with different factors after a 2-week placebo treatment. There was no statistical difference in the frequency of likely active placebo effect either in the subgroups of gender and age. There were numerically more patients with active placebo effect in the relatively shorter disease duration subgroup (<4 months [76%]), higher initial VAS subgroup (>60 mm [90%]), and worse initial NDI subgroup (>24 [72%]) compared with the gender and age subgroup.

**TABLE 2:** Summary and analysis of likely active placebo effect.

	Total	Trial A	Trial B	Model (75.7%)		
	(*N* = 162) *n* (%)/Percentiles	(*N*= 102) *n* (%)/Percentiles	(*N* = 60) n (%)/Percentiles	OR	95% CI	*p*-Value
Active placebo effect	60%	53%	73%	-	-	-
Sex (Male)	41 [25%]	26 [25%]	15 [25%]	0.663	0.290–1.518	0.331
Age (years)	52.5 (41,60) [40%]	52 (42,60) [40%]	55 (39,59) [42%]	0.990	0.961–1.021	0.540
Duration (months)	4 (2,8) [24%]	5 (2,10) [22%]	3 (2,4) [35%]	0.890	0.764–0.989	0.038^*^
VAS (mm)	60 (43,70) [90%]	60 (43,66) [90%]	59 (43,72.5) [88%]	1.063	1.037–1.090	0.000^*^
NDI	24 (18,30) [72%]	21 (17,27) [69%]	28 (22,37.88) [80%]	1.013	1.047–1.073	0.042^*^

^*^, at the p<0.01 level (two-tailed), the correlation is significant.

**FIGURE 2:**
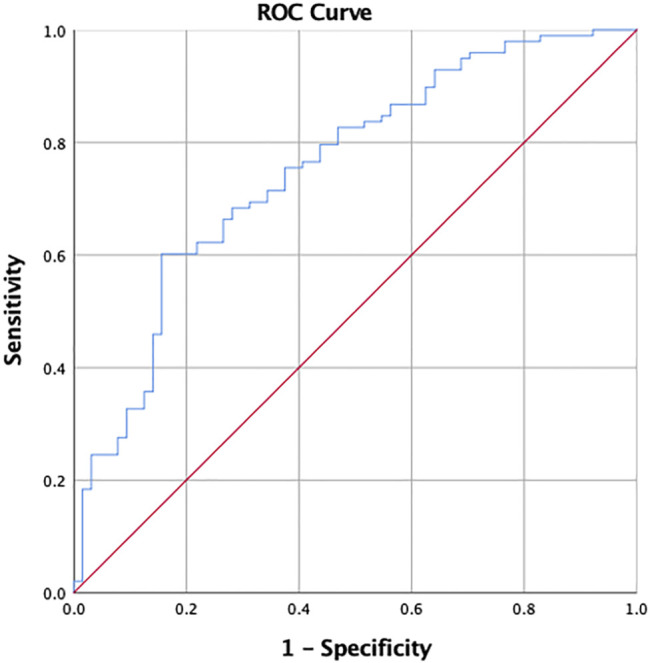
ROC curve of model fitting for active placebo effect prediction of goodness-of-fit test.

A series of the likelihood ratio is derived from the predictive model as above, which provides pairs of sensitivity and specificity from these factors. An ROC curve was established to assess the model-data fit, which showed an area under the curve of 0.755 and a 95% confidence interval of 0.677–0.830 (Figure 2). Thus, the accuracy of this predictive model is quite well.

Hosmer–Lemeshow test was used to assess the goodness of fit of the model, which showed that the chi-square value is 9.838 with a degree of freedom of 8 (*p* = 0.277, not less than the test level of 0.05 (Table 3). Thus, it is considered the goodness of fit of the model is high, which means that the information in the current data has been fully extracted.

**TABLE 3:** Contingency table for Hosmer–Lemeshow test.

	Non-Placebo Effect		Active Placebo Effect		
	Observed	Expected	Observed	Expected	Total
1	14	12.712	2	3.288	16
2	9	10.339	7	5.661	16
3	8	9.166	8	6.834	16
4	9	7.44	7	8.56	16
5	7	6.502	9	9.498	16
6	7	5.443	9	10.557	16
7	1	4.504	15	11.496	16
8	3	3.547	13	12.453	16
9	5	2.57	11	13.43	16
10	1	1.778	17	16.222	18

### Persistent of Active Placebo Response

To further investigate how long the active placebo effect lasts, the changing trend of VAS and NDI were analyzed after the second 2 weeks. In terms of pain relief and function improvement, participants who show an active placebo effect after 2 weeks had significantly lower VAS scores, while there was no significant difference in NDI improvement between the two groups (Shown in Table 4).

**TABLE 4:** Summary and comparison of VAS and NDI at all the placebo-treatment visit.

		Day 0	Week 2	Week 4	*p*-value
VAS	Active placebo effect	62.389 (58.019–66.759)^*^	46.796 (2.221–42.426)^*^	35.185 (30.815–39.555)^*^	0.000^**^
	Non-placebo effect	48.104 (43.469–52.740)^*^	40.938 (36.302–45.573)^*^	40.292 (35.656–44.927)^*^	0.000^**^
	*p-value*	0.008	0.008	0.008	
NDI	Placebo effect	22.259 (20.742–23.776)	12.611 (11.094–14.128)	11.296 (9.779–12.813)	0.000^**^
	Non-placebo effect	22.729 (21.120–24.338)	12.208 (10.599–13.817)	9.604 (7.995–11.213)	0.000^**^
	*p-value*	0.404	0.404	0.404	

^*^, at the p<0.01 level (two-tailed), the difference between groups is significant;

^**^, at the p<0.01 level (two-tailed), the difference between time windows is significant;

## Discussion

This study pooled data from two placebo-controlled randomized trials on the treatment of patients with neck pain, a matching placebo for the control of the Qishe pill (a proprietary Chinese medicine), and ICT for the control of Shi-style manipulation (a traditional Chinese medicine manipulation). Both interventions have been identified as placebo treatments in previous studies (Harrington. 1997; Finniss, 2013; Benedetti, 2014).

In these two RCTs for neck pain, we found that some patients with short disease duration may overscore their pain severity, who may also report a large improvement in pain and function, even in the placebo group. Extracted and re-divided into two groups based on whether there was a substantial improvement in pain after 2 weeks to define whether there was a “placebo effect”, using the subjects’ baseline information as a potential risk factor, a logistic regression analysis was used to explore the risk factors causing different placebo response, and probability of placebo effect was predicted based on these risk factors.

This study presented a reasonable and objective method to quantify and evaluate the perception and the expectation in placebo therapy, which showed that pain severity and disease duration are the two most important factors in placebo responses. Neck pain patients with shorter disease duration may be hard to describe their perception of pain, which may probably overscore in their first self-report and easily decrease the pain score after a 2-week placebo treatment with their expectations. On this basis, in the recruitment process, it is necessary to exclude neck pain patients with shorter disease duration, who present extremely high self-reported pain. If patients have finished all the treatment and follow-ups, in the statistic process, some weighted conversion from the probability of placebo response may take place of a simple “A-B” efficacy difference between two groups.

These results on the probability of placebo effect interpreted effect on different pain perception, tolerance, and expectation, which may be related to individual emotion status and disease duration. Patients’ perceptions of the treatment may directly influence the outcome, which may be influenced by treatment description (Bausell et al., 2005) and verbal suggestions (Bingel et al., 2011). Studies of patients with chronic pain from neuropathic pain (Kupers et al., 2007; Petersen et al., 2012) reported some similar involvement of expectations in emotions (Vase et al., 2005; Kupers et al., 2007; Skyt et al., 2018). Other studies have shown that endogenous opioid and non-opioid mechanisms are associated with placebo analgesia (Peciña and Zubieta. 2015). When a patient takes a placebo therapy, positive expectations and conditioning activate these neurobiological systems and induce a series of physiological changes that are maintained by reward learning, leading to analgesia and improvement of psychological status. This reward mechanism may be weakened with disease lasting and expectations decreasing. The placebo effect can be enhanced or abolished through active topical or opioid analgesia from positive or negative expectations. However, patients’ expectations toward treatment outcomes in RCTs are usually not assessed.

Our study has limitations, first, the participants we investigated are only patients with neck pain; second, the sample size is moderate to conduct this analysis. Despite the limitations, these studies provide insight into the potential placebo effect in patients with neck pain. In order to verify the accuracy of placebo effect detection, a more accurate objective measurement of pain and its related brain functional MRI will be used for further investigation, which may reveal specific processes of analgesia (pain perception, pain tolerance, or pain induction) with accurate evidence (Amanzio et al., 2013; Jubb and Bensing. 2013; Buchel et al., 2014). Also, future clinical trials will need to take into account patients’ disease duration and self-reported pain severity to screen participants with high expectations for pain relief.

## Conclusion

In summary, a logistic regression analysis has shown that disease duration of neck pain is the most risk to present an excessive placebo effect in management. Neck pain patients with shorter disease duration are more likely to overscore their pain severity, because of their less experience in pain perception, tolerance, and analgesia expectation.

## Data Availability

The original contributions presented in the study are included in the article/Supplementary Material, further inquiries can be directed to the corresponding authors.
